# Upper Airway Flow Dynamics in Obstructive Sleep Apnea Patients with Various Apnea-Hypopnea Index

**DOI:** 10.3390/life12071080

**Published:** 2022-07-19

**Authors:** Shengmao Lin, Thyagaseely Sheela Premaraj, Peshala T. Gamage, Pengfei Dong, Sundaralingam Premaraj, Linxia Gu

**Affiliations:** 1 School of Civil Engineering and Architecture, Xiamen University of Technology, Xiamen 361024, China; linshengmao@gmail.com; 2 College of Dental Medicine, Nova Southeastern University, Fort Lauderdale, FL 33328, USA; tpremara@nova.edu (T.S.P.); spremara@nova.edu (S.P.); 3 Department of Biomedical and Chemical Engineering and Sciences, Florida Institute of Technology, Melbourne, FL 32901, USA; pdong@fit.edu

**Keywords:** obstructive sleep apnea (OSA), apnea-hypopnea index (AHI), airflow dynamics

## Abstract

Background and aim: This study evaluates the upper airway flow characteristics, anatomical features and analyzes their correlations with AHI in patients with varied degrees of OSA severity seeking for discernments of the underlying pathophysiological profile. Materials and Methods: Patient-specific computational fluid dynamics models were reconstructed from high-resolution cone-beam computed tomography images for 4 OSA patients classified as minimal, mild, moderate, and severe according to AHI. Results: The parameters, minimal cross-sectional area (MCA), and the pharyngeal airway volume did not show clear correlations with the OSA severity defined according to AHI. No correlations were found between the classically defined resistance of the airway in terms of pressure drop and AHI. The flow analysis further showed that the fluid mechanisms likely to cause airway collapse are associated with the degree of narrowing in the pharyngeal airway rather than AHI. Results also suggested that some patients classified as severe OSA according to the AHI can show less susceptibility to airway collapse than patients with relatively lower AHI values and vice versa. Conclusions: The relative contribution of anatomical and non-anatomical causes to the OSA severity can significantly vary between patients. AHI alone is inadequate to be used as a marker of the pathophysiological profile of OSA. Combining airflow analysis with AHI in diagnosing OSA severity may provide additional details about the underlying pathophysiology, subsequently improving the individualized clinical outcomes.

## 1. Introduction

Obstructive sleep apnea (OSA) is a common sleep-related disorder due to repetitive collapse of the upper airway. OSA is seen in 9–38% of the overall population and its prevalence significantly increases with age [1]. OSA is commonly linked with clinical symptoms such as snoring, and daytime sleeplessness. Clinical studies have associated OSAS with obesity, cardiac disorders, neurocognitive disease, and cancer [2,3]. Polysomnography is the gold standard method for diagnosing OSA and its severity. It records the occurrences of apneas (pauses in breathing) and/or hypopneas (periods of shallow breathing) overnight. Specifically, the apnea index (AI) was adopted [4] to monitor the number of apneas per hour of sleep. The apnea-hypopnea index (AHI) represents the mean number of apneas and hypopneas per hour of sleep since non-apneic breathing disturbances are also associated with symptoms of OSA that can exist in the total absence of apneas [5]. Currently, AHI serves as the most common measure to diagnose the severity of OSA.

The American Academy of Sleep Medicine recommends continuous positive airway pressure (CPAP) as the treatment for all severities of OSA [6]. It suggests the use of oral appliances in patients suffering from snoring, mild-to-moderate OSA, or severe OSA if CPAP has failed [2] where the severity is determined based on AHI. The oral appliances are designed to keep the airway open for adequate ventilation during sleep. Although CPAP is extremely effective, 25% to 50% of patients with OSA have difficulty tolerating CPAP [7], and adherence to this treatment is poor [8,9]. Moreover, cases where AHI is severe have been treated successfully with oral appliances [10]. The individual variation in response to CPAP therapy is the challenge in treating patients indiscriminately with a single treatment option and expecting the best outcome. Focusing solely on the number of respiratory events during sleep (i.e., AHI) may oversimplify the diagnosis and treatment of OSA in many patients, consequently leading to unexpected clinical outcomes [11,12].

The treatment outcomes of OSA could be enhanced with a better understanding of the patient-specific pathophysiological causes. Both anatomical and non-anatomical factors can contribute to the OSA severity, and these factors are varying among OSA patients [12]. The study of airflow in OSA patients can provide details about how the airway anatomy affects the airflow dynamics causing the airway to collapse. This, in turn, will help to identify the anatomical factors that contribute to the OSA severity on patient-specific basis. Computational simulations have been widely utilized to provide an invaluable perception that complement and guide experimental measurements [13,14,15,16,17,18,19,20]. The potential of computational fluid dynamics (CFD) in predicting clinical outcomes and assessing the diseased conditions in human upper airways has been previously demonstrated [21,22,23,24,25,26,27]. Some of these studies characterized upper airway flow dynamics under diseased conditions [23,25,26] whereas others have evaluated the clinical intervention outcomes by comparing the pre- and post-intervention conditions [21,24,27]. Specifically, our previous work has constructed CFD models based on cone beam computed tomography (CBCT) images of patients to quantitatively evaluate the airflow changes that occurred within the pharyngeal airway space. We observed that the flow vorticity has a better correlation with the airway flow resistance than the dimensions [28]. Studies integrating the AHI with the airflow dynamics [26,29,30] are scarce.

In this study, we used CFD to analyze the upper airway flow dynamics of patients with varied degree of disease severity classified according to AHI. The constructed 3D airway geometries included both nasal passages and pharyngeal airway sections. We specifically focus on accessing the flow mechanisms plausibly causing airway occlusion in OSA patients. The localized distribution of the pressure, velocity, aerodynamic forces, and flow resistances were analyzed. Here, we determined to explore the common indicators associated with OSA, the AHI, minimum cross-sectional area (MCA), and the shape and size of the upper airway to air flow mechanics to evaluate whether any possible correlations exist between patient’s airway characteristics and airflow dynamics. Flow analysis will help to relate anatomical factors that contribute to the level of OSA severity assessed by AHI. The correlations between AHI and flow parameters will help determine the relative contribution of anatomical and non-anatomical factors to OSA severity. The objective of the study is to evaluate the upper airway flow characteristics and anatomical features and analyze their correlations with AHI in patients with varied degree of OSA severity seeking discernments on the underlying pathophysiological profile. This would be of interest to the clinician in identifying patients for successful CPAP therapy and reducing the economic burden for the patients and the healthcare system. The long-term goal is to find a patient identifier to determine the suitability for the most efficacious treatment modality CPAP [31].

## 2. Materials and Methods

### 2.1. Subjects

Upper airway geometries extracted from four OSA patients were employed in the study after obtaining Institutional Review Board approval from the University of Nebraska Medical Center (IRB approval # 460-15-EX). These patients were selected on the basis that they had varying degrees of disease severity, as shown in Table 1.

### 2.2. Image Analysis

All radiographic scans were taken with the i-CAT 17-19 CBCT (Imaging Sciences International LLC, Hatfield, PA, USA) with a field of view of 23 × 17 cm and a voxel size of 0.3 mm by a single individual. Subjects were instructed to stand upright and look straight ahead as if looking into a mirror. They were adjusted to have Frankfort horizontal plane parallel to the floor, if possible. Patients in the upright position were asked to breathe normally and have their tongue rest on the floor of the mouth at the time of scan acquisition. Informed consent was obtained from all subjects.

**Figure 1:**
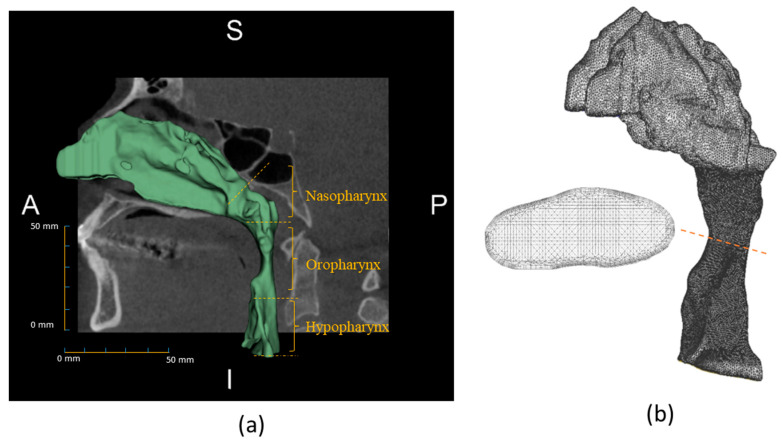
(**a**) Extracted airway geometry from cone-beam CT images with specified pharyngeal sections. (**b**) The meshed volume of the airway with a cross-sectional view at the location is denoted by the dashed line.

Patient-specific CFD models were reconstructed from CBCT images using an open source software: 3D slicer [32]. Here, the air volume was first isolated by specifying a threshold range from −1000 to −400 based on the grayscale value of the images. Then, the pharynx volume was separated by cropping the region of interest. Figure 1a shows an extracted airway geometry. The morphological characteristics of extracted airways are summarized in Table 2.

### 2.3. Airflow Simulation

The airflow was simulated using the commercial CFD software, ANSYS Fluent (ANSYS Inc., Canonsburg, PA, USA). As the airflow timescale is much smaller than the breathing cycle duration, a quasi-static flow was assumed [33]. The standard steady RANS k-w turbulence model was used to capture the turbulence generated in the upper airway. RANS k-w turbulence model has previously shown better agreement with the flow measurements in human upper airways compared to other common models such as k-epsilon, Spalart-Allmaras one-equation model, and even k-w SST model [34]. Whereas Large Eddy Simulation (LES) can capture small-scale flow structures at the expense of computational complexity, studies have shown that same or better agreement with the experimental results is expected when LES is used instead of k-w [35]. The inspiratory flow was simulated by imposing a flowrate of 300 mL/s at the nasal inlet [28]. The pressure at the pharynx outlet was set to 0 Pa as a reference pressure. A turbulence intensity of 10% was assumed at the inlet [30]. A non-slip boundary condition was specified at the airway wall.

The reconstructed airway geometry in STL format was imported to ANSYS ICEM CFD where an unstructured tetrahedral mesh volume was generated for the CFD fluid domain. Grid independence studies were conducted (for each patient geometry) by considering the maximum pressure, maximum velocity, and velocity distribution at the narrowest cross-section of the geometry. The mesh was selected when the change is considered parameters was less than 0.5% when the mesh is iteratively fined. The final mesh contained ~2,000,000 elements, which had a maximum size of 1 mm and minimum size of 0.2 mm with a growth rate of 1.2. A prism layer mesh consists of 4 layers with an initial thickness of 0.05 mm and a growth rate of 1.3 was used to accurately capture near-wall flow dynamics.

## 3. Results and Discussion

### 3.1. Airway Dimension Characteristics

Geometric characteristics, including pharyngeal volume and minimum cross-sectional area (MCA) of the airways are shown in Table 2. The MCA is 69.97 mm^2^, 49.74 mm^2^, 133.9 mm^2^, and 67.2 mm^2^, for patients 1 to 4, respectively. The low MCA values are expected to cause high resistance to airflow, thus, associated with increased OSA severity. Significance differences in MCA measurement between OSA and non-OSA groups have been reported [30,36]. Previous analysis of CBCT data has shown a significant difference in the minimum pharyngeal area between the OSA patients and non-OSA subjects aligning with their MCA measurements of (64.7 ± 42.7) mm^2^ and (86.1 ± 42.3) mm^2^, respectively [36]. The MCA measurements in our study did not show a correlation with the AHI index. Although the AHI index of patient 3 suggests moderate OSA severity, the relatively larger MCA suggested less severe airway obstruction. Moreover, patient 1 had a much smaller MCA compared to patient 3, despite being classified as having minimal OSA severity based on AHI. A low correlation between the pharyngeal area and AHI (in OSA patients) is also reported in previous work [37]. These results suggest that OSA severity cannot be solely associated with airway geometry.

### 3.2. Airway Flow Analysis

The velopharynx (i.e., the narrowest section of the pharyngeal airway) of OSA patients obstructs the airflow and is considered the most susceptible region to be collapsed during breathing [38]. The increased airflow speed and low intraluminal airway pressures near the velopharynx are identified as key factors that contribute to the development of OSA [39]. The constriction in the pharyngeal airway can cause significant alterations in fluid flow dynamics near the airway wall. Hence, the analysis of airflow dynamics can provide insights into the severity of OSA from an aerodynamic perspective. Accordingly, to assess the magnitude of plausible flow mechanisms that can cause OSA, we investigated the pressure drops, velocity distributions, pressure forces, and flow resistances of the airway geometries. It should be noted that the flow in all patients is driven by the imposed constant flowrate across the airway geometry, thus, the flow dynamic changes solely depend on the geometric configuration of the airways.

**Figure 2:**
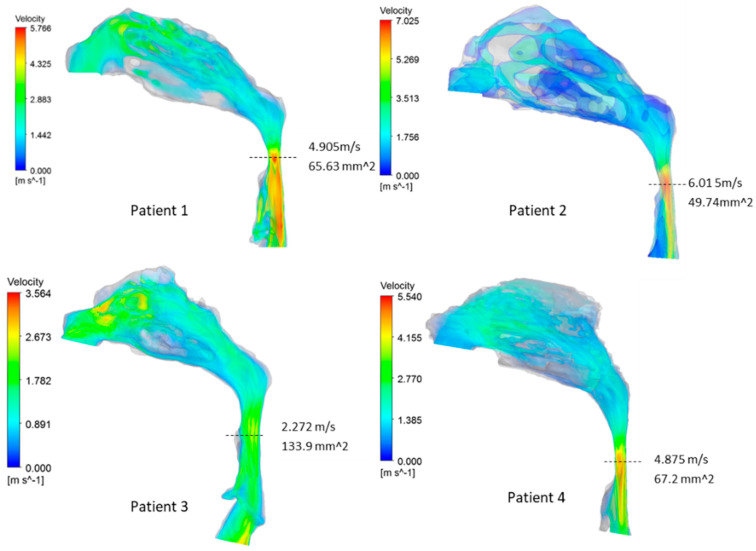
Velocity distributions in airways. Minimum cross-sectional areas are denoted with vertical dashed lines.

**Figure 3:**
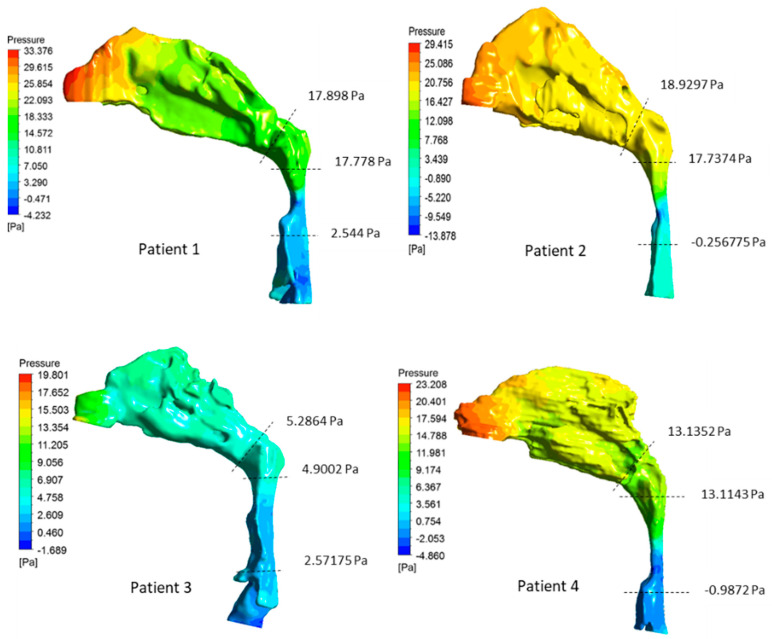
Pressure distribution on the airway wall. Pressure levels at the nasopharynx and orophaynx boundaries are specified with dashed lines.

Figure 2 and Figure 3 show the velocity and pressure distribution in the airways, respectively. For all patients, the minimum cross-sectional area was located in the oropharynx region where the maximum velocity was observed. The maximum velocities were in the range of 2.2–6.01 m/s, which is comparable to velocities observed with OSA patients in other computational studies [30,33]. For patients 1, 2, and 4, a high-velocity jet was observed, which originated at the narrowing in the oropharynx region. This jet flow structure continued through the posterior region of the hypopharynx. In contrast to other patients, a significant narrowing in the pharyngeal region was not seen in patient 3 airway geometry. Hence, a relatively smoother airflow was observed without a distinct high-velocity jet flow that extends through the hypopharynx region.

Correlated with the velocity distribution, a significant pressure drop across the narrowing in the oropharynx region was seen in patients 1, 2, and 4. Due to the high acceleration of the airflow at the narrowing of these patients, the local pressure in the narrowest area became lower than the outlet pressure. Increased pressure drops in OSA patient airways have been observed in the region from choanae to the maximum narrowing where the pharynx is constricted by adenoids and tonsils [40]. In contrast to other patients, the pressure drop across the nasal passage of patient 3’s airway was higher than the pressure drop across the pharyngeal region.

The jet flow structure generated at the constriction of the oropharynx region is referred to as “pharyngeal jet,” which is often observed in OSA patient airway geometries [38]. The pharyngeal jet causes a high-pressure drop across the narrow region and generates large variations of pressure and velocity downstream of the narrowing.

**Figure 4:**
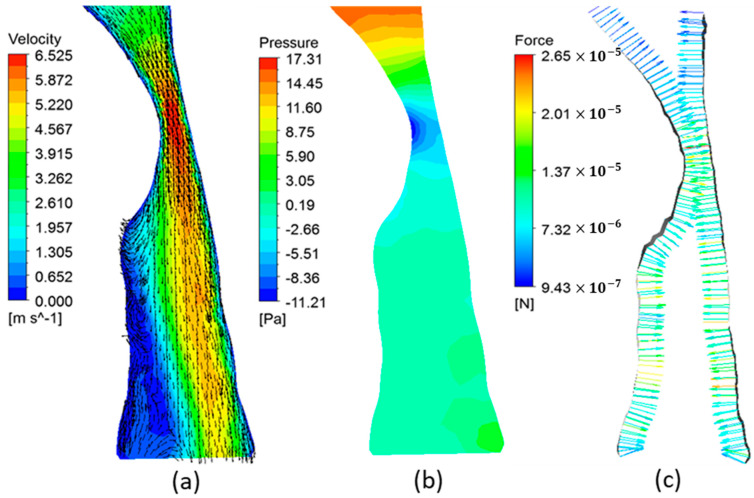
(**a**) The velocity contours and vectors, (**b**) pressure contours, and (**c**) pressure forces on airway wall at the mid-sagittal plane of patient 2 airway.

**Figure 5:**
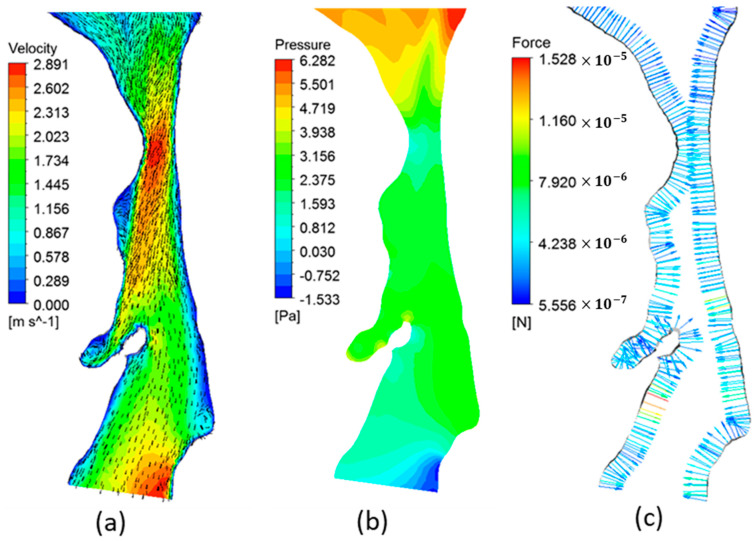
(**a**) The velocity contours and vectors, (**b**) pressure contours, and (**c**) pressure forces on the airway wall at the mid-sagittal plane of patient 3 airway.

Figure 4 and Figure 5 illustrate the variations of velocity, pressure, and fluid pressure forces at the mid-sagittal cross-section of the airway of patient 2 and patient 3, respectively. Patients 1, 2, and 4 showed a similar flow pattern. Thus, the corresponding results for patient 1 and 4 are not shown. In realistic conditions, during inspiration, the pressure at the nasal inlets is equal to the atmospheric pressure and the pressure inside the airways is lower than the atmospheric pressure. Hence, the pressure forces are calculated assuming a zero-gauge pressure at the nasal inlets. As seen in Figure 4a, the pharyngeal jet generated in patient 2 airway extends through the hypopharynx region directed toward the posterior wall. High flow circulations were observed downstream of the narrowing created by the flow separation caused by the high-velocity jet. The energy losses due to flow separation contribute to a further increase in the pressure drop in the airway. The pressure distribution results of patient 2 (shown in Figure 4b) indicated the minimum pressure located at the narrowest section on the anterior side of the wall, which can lead to a stronger surface suction force on the soft palate [33]. Furthermore, due to high inertia and flow separations, the pressure downstream the narrowing became less than the pressure at the outlet (i.e., negative gauge pressure). This low pressure can create strong suction forces on the surface of the airway, which is shown in Figure 4c. For OSA patients, the relaxed pharynx muscles during sleep can act as a collapsible tube. Combined with aerodynamic suction forces, the narrowed airway can eventually result in a collapse [33,38]. The upper airway collapse due to the low pressure generated at the oropharynx region due to the pharyngeal jet flow is previously observed [41].

The velocity distribution observed in patient 3 (shown in Figure 5a) was different from other patients. Due to the larger luminal area, the maximum velocity at the narrowing was much smaller than other patients. Moreover, the pharyngeal jet disappeared downstream inside the oropharynx region. In addition, in contrast to other patients, the pharyngeal jet was directed towards the anterior wall. It is likely that the distribution and direction of the pharyngeal jet strongly depend on the location of the narrowing as well as the geometry of the pharynx. Hence, in addition to the severity of the narrowing, the aerodynamic forces on the airway wall can significantly change with the patient-specific airway characteristics such as the location of the narrowing and the shape of the pharyngeal airway. In contrast to other patients, pressure levels at the constricted area of patient 3 were higher than the outlet pressure (as seen in Figure 5b). Ina ddition, the pressure forces on the airway wall were relatively weaker and the highest force was observed in the hypopharynx region (as seen in Figure 5c). From an aerodynamic perspective, this force distribution suggests that patient 3 is less susceptible to the possibility of airway collapse compared to other patients.

Increased flow resistance in the upper airway results in increased breathing effort. OSA patients found to have increased pharyngeal flow resistances caused by the narrowing generally located in the oropharynx region [30,33]. The flow resistance of the pharyngeal section ($R_{Pharynx})$ and oropharynx section ($R_{Oroharynx})$ was evaluated as the ratio between the pressure drop and flowrate across the respective sections. The calculated flow resistances are shown in Table 2. The flow resistance increased with the decrement in the minimum cross-sectional area. The highest flow resistance of 63.09 Pa·s·L^−1^ was observed in patient 2, followed by resistances of 59.66 Pa·s·L^−1^ and 47.01 Pa·s·L^−1^ observed in patients 1 and 4, respectively. In these patients, the flow resistance across the oropharynx section contributed to over 85% of the pharyngeal flow resistance. Patient 3 had the minimum airflow resistance where the oropharynx section contributed to only 44% of the total pharyngeal flow resistance and higher resistance was observed in the hypopharynx region. The focus of the study was to analyze airway characteristics and flow dynamics with the OSA severity. Thus, only OSA patients were considered. Non-OSA patients are expected to have significantly lower AHI (<<5) as well as a significantly larger minimum cross-sectional area compared to OSA patients. Hence, due to the low level of constriction, benign flow conditions are expected in healthy people.

**Table 2:** Flow resistances and morphological characteristics of the airways.

Patient	Pharyngeal Volume (cm^3^)	Min. Cross-Sectional Area (mm^2^)	$R_{oropharynx}$ (Pa·s·L^−1^)	$R_{pharynx}$ (Pa·s·L^−1^)	AHI
1	10.49	69.97	50.78	59.66	4
2	10.63	49.74	59.98	63.09	10.4
3	18.67	133.9	7.76	17.62	20.3
4	14.33	67.2	43.78	47.01	50.1

### 3.3. AHI and OSA Severity

Initially, the cut-off value of at least 30 apneic events in an overnight 7 h sleep period (i.e., AI > 5) was suggested to differentiate the diseased condition [4]. This value was derived from a limited cohort of sleep apnea patients compared with 20 control subjects and subsequently became a norm in clinical trials. However, this criterion has not been reproduced or validated since then [42]. Later in 1999, the American Academy of Sleep Medicine introduced a formal classification of disease severity that AHI ≥ 15 per h represents “moderate disease”, AHI ≥ 30 per hour represents “severe disease” and AHI values 15 > AHI ≥ 5 per represents “mild disease” [43].

Despite its wide acceptance, the use of arbitrary AHI cut-off values for separating diseased conditions from normalcy has been criticized [44] and shown that AHI can imply false positives in predicting OSA [45]. Studies evaluating correlations between AHI and OSA severity features, such as hypersomnolence and hypertension, have shown weak and negative correlation coefficients [46]. The technical differences in the sensors detecting the respiratory disturbances and differences in hypopnea definitions can also influence the AHI measurements and assessment of OSA severity [42]. Studies have emphasized the severity categories implied by AHI cut-off values alone are misleading and arbitrary for clinical decision-making [47]. Focusing solely on the number of respiratory events during sleep (i.e., AHI) may oversimplify the diagnosis and treatment of OSA in many patients, consequently leading to unexpected clinical outcomes [12].

### 3.4. Airway Flow Dynamics and OSA Severity

Our results showed that the extent of the fluid dynamics mechanisms that are likely to cause airway collapse is closely associated with the degree of narrowing in the pharyngeal airway. However, the clinical measure of OSA severity (i.e., AHI) did not show clear correlations with the airflow mechanics or the degree of constriction. For instance, flow dynamics in patient 3 airway showed less susceptibility to airway collapse than patients with relatively lower AHI values. Despite being classified as minimal and mild OSA severity, patients 1 and 2 showed significant airway narrowing and more tendency toward airway collapse from a fluid dynamics perspective. This may be explained by the fact that OSA severity measured by AHI is affected by both anatomical and non-anatomical factors. Although the narrowed pharyngeal airway geometry can cause increased closing pressures that affect the ability of the upper airway dilating muscles to maintain airway patency, non-anatomical traits such as genioglossus muscle responsiveness, and respiratory control stability and arousal threshold can play a major role in airway collapse [48].

No correlations were found between the classically defined resistance of the airway in terms of pressure drop and AHI. The flow analysis in this work showed that the airflow-caused airway collapse is associated with the degree of narrowing in the pharyngeal airway rather than AHI. The severity of such an OSA condition is likely to be dominated by the non-anatomical pathophysiological factors than the airway anatomical factors. This raises questions as to whether AHI is an accurate measurement of OSA severity and whether it can be used solely to decide on a clinical treatment strategy. Consideration of both flow dynamics conditions and AHI may provide a more accurate diagnosis of the disease, allowing the selection of a better patient-specific treatment strategy.

### 3.5. Study Limitations

The limitation of the airflow dynamics analysis is the parameters set forth for the analysis may not be truly reflective of the natural conditions. Finding the mechanical and physiological properties of the airway and the air pressure at different locations that could truly reflect the true nature would be of importance for future studies.

OSA severity can be influenced by airway wall compliance, which was not included in the simulations. The pharyngeal wall is known to deform during respiration, which, in turn, alters the flow dynamics [49]. This phenomenon can be significant during inspiration when airway pressure decreases causing further reduction in the cross-sectional area [50]. Although a patient may have low airflow resistance at low flowrates, it’s possible to have higher resistances at higher flowrates due to the compliance of airway wall, which can cause airway collapse. In addition, the characteristics of surrounding anatomical structures of the airway wall can also affect wall compliance. For instance, the accumulation of soft tissues and fat would increase the tissue pressure causing the airway more prone to collapse [50]. Fluid structure interaction (FSI) modeling incorporating medical imaging can be helpful in studying the relevance of anatomical structures to airway collapse.

The air flow resistance was conventionally defined as the ratio between the pressure drop and the flowrate. This measure is more suitable for comparing the flow resistance between different airway geometries if the pressure drop is linearly related to the flowrate. However, for the flowrates 200–500 mL/s the upper airway flow is identified to be transitioning from laminar to turbulent [50], in which the pressure is no longer linearly related to the flowrate. The pressure-flow relationship in the transition region can be accurately described using a quadratic relationship:

$$\nabla P=k_{1}V+k_{2}V^{2}$$

where $k_{1}$ is associated with the pressure drop in the laminar flow regime and $k_{2}$ is associated with the pressure drop in the turbulent flow [49]. This study imposed a flowrate of 300 mL/s for all the patients and a similar approach was used in previous studies [28]. The significant differences in airway volumes suggest that certain patients are likely to breathe more air subsequently having a relatively higher flowrate. Thus, employing patient-specific flowrates will provide a more accurate comparison of airflow resistance to assess the OSA severity.

Analysis of airflow with more patient data categorized into homogeneous groups based on age, sex, and BMI is required to confirm the observations in this study. However, the study shows the possibility of airflow conditions (i.e., resistance, pressure drop) and anatomical features not correlating with the severity measured by AHI. It can be expected for patients with less severe flow conditions to have very high AHI values and vice versa due to the relative contribution of anatomical and non-anatomical factors to the severity of OSA.

## 4. Conclusions

The airflow dynamics were analyzed in upper airway geometries derived from four OSA patients classified as minimal, mild, moderate, and severe conditions according to AHI. The flow dynamics results showed that a low-pressure region is generated downstream of the airway narrowing, which exerts aerodynamic suction forces on the lumen wall. These forces increased with the degree of narrowing whereas force distribution changed based on the location and the shape of the airway geometry. No clear correlations were found between the airflow dynamics, airway geometry, and AHI. In fact, the results suggested that an airway with flow dynamics indicating benign airflow dynamics does not always indicate low OSA severity (i.e., low AHI) and vice versa. This indicated the importance of identifying the pathophysiological profile of OSA as the contribution of anatomical and non-anatomical features to the OSA severity can vary. Thus, AHI alone might not a good option to be used as a marker of the pathophysiological profile of OSA. However, the upper airway flow dynamics are closely associated with the anatomical factors of OSA, which can potentially serve as a method to uncover more details about the underlying pathophysiology of an OSA patient. For instance, a patient demonstrating a benign airflow profile (i.e., relatively low airflow resistance and weaker aerodynamic suction forces on the wall) with a severe AHI index (>30) may indicate a more significant effect from non-anatomical factors of the patient such as muscle responsiveness, arousal threshold, and respiratory control stability. Another patient with severe AHI with airflow dynamics indicating a high tendency to airway collapse may suggest a higher effect from airway narrowing. Thus, the combined analysis of features derived from airflow and AHI has the potential for a more accurate diagnosis of patient-specific pathophysiology profile. Such diagnostic procedure will help to improve the individualized treatment of OSA for better clinical outcomes.

## Figures and Tables

**Table 1:** Patient demographics and AHI.

Patient	Gender	Age	BMI	AHI	OSA Severity
1	Female	55	23.6	4	None/Minimal: AHI < 5
2	Female	57	35.3	10.4	Mild: AHI 5 < AHI < 15
3	Female	65	25.7	20.3	Moderate: 15 < AHI < 30
4	Male	46	36.4	50.1	Severe: 30 ≤ AHI
